# Health Tract, No. 40

**Published:** 1861-11

**Authors:** 


### HEALTH TRACT, No. 40.

From Hall's Journal of Health, $1 a year, New-York.

### WHAT TO EAT, AND WHEN.

When a piece of land is exhausted of the element which is the principal ingredient of a certain crop, that ingredient must be supplied, or the crop will fail in quantity and in quality; hence the thrifty farmer ascertains the wants of the soil, and supplies it with the needed manure every year. The human body is exhausted of its elements day by day, and day by day must these elements be supplied by what we eat and drink; but the required proportion of these elements changes with the seasons, with the temperature of the weather, and he who eats the same in quantity and quality in July as at Christmas, will die in a month, because the adult eats for two reasons—to warm and to nourish. All food contains two chief principles: Carbon, to keep from freezing; Nitrogen, to keep from famishing. The proportion of these elements varies with the food. Those who work a great deal, require a great deal of nourishment, of nitrogen, for it is the flesh-forming principle. Those who are exposed a great deal to the cold should eat the carbonaceous, the heat-supplying food. Butter and fat are three fourths carbon; vegetables have but little, berries none. Hence Greenlanders in their icy homes luxuriate in blubber and whale-oil, while the people of the sunny South revel in oranges and bananas, on the plantain and the peach, on dates and figs, on lemons, tamarinds, pine-apples, etc. We who live in latitudes between, are permitted the diet of the Polar Sea and the tropics, in their season. A wise man will take but little carbonaceous food on a suddenly hot day; but if suddenly cold, it is best for him to eat more of fuel-making food. An infinite number of fevers and of colds would be avoided if timely attention were paid to these things. By the aid of these statements, the following tables may be used to great advantage, showing the amount of carbon, or heat-forming principle, in several articles of food. There is not one per cent of nitrogen, or flesh-forming principle, in fruits, berries, and the more common vegetables. Meats have about fifteen per cent. The meats average twenty-five per cent of nutriment, that is, including both carbon and nitrogen. Of all meats, mutton is the most nutritious—thirty per cent; fish least, twenty per cent. Of all vegetables, white beans are the most nutritious, ninety-five per cent; wheat-flour, ninety per cent; turnips, the least, five per cent. Of fruits, plums are the most nutritious, thirty per cent; apples, seventeen; melons and cucumbers, three, the rest being mere water and waste. The more waste, the more open the bowels are.

| | Percentage of Carbon. | | Percentage of Carbon. | | Percentage of Carbon. |
|---|---|---|---|---|---|
| Apricots, | 0 | Potatoes, | 11 | Wheat Bread, | 40 |
| Berries, | 0 | Lean Meat, | 13 | Sugar, | 42 |
| Cherries, | 0 | Rye Bread, | 31 | Apples, | 45 |
| Currants, | 0 | Gum Arabic, | 36 | Meats, Fat, | 53 |
| Turnips, | 3 | Arrow-Root, | 36 | Butter, | 65 |
| Artichokes, | 9 | Green Peas, | 36 | Soup, | 75 |
| Blood, | 10 | Starch, | 37 | Lard, | 80 |
| Milk, | 10 | Lentils, | 37 | Beans, | 88 |

